# Identification of Copy Number Variation Among Nonsyndromic Cleft Lip and or Without Cleft Palate With Hypodontia: A Genome-Wide Association Study

**DOI:** 10.3389/fphys.2021.637306

**Published:** 2021-02-26

**Authors:** Norliana Ghazali, Normastura Abd Rahman, Azlina Ahmad, Sarina Sulong, Thirumulu Ponnuraj Kannan

**Affiliations:** ^1^School of Dental Sciences, Universiti Sains Malaysia, Kubang Kerian, Malaysia; ^2^Human Genome Centre, School of Medical Sciences, Universiti Sains Malaysia, Kubang Kerian, Malaysia

**Keywords:** cleft lip, cleft palate, hypodontia, DNA copy number variations, genome-wide association study

## Abstract

Nonsyndromic cleft lip and or without cleft palate (NSCL/P) with the hypodontia is a common developmental abnormality in humans and animals. This study identified the genetic aberration involved in both NSCL/P and hypodontia pathogenesis. A cross-sectional study using genome-wide study copy number variation-targeted CytoScan 750K array carried out on salivary samples from 61 NSCL/P and 20 noncleft with and without hypodontia Malay subjects aged 7–13 years old. Copy number variations (CNVs) of *SKI* and fragile histidine triad (*FHIT*) were identified in NSCL/P and noncleft children using quantitative polymerase chain reaction (qPCR) as a validation analysis. Copy number calculated (CNC) for each gene determined with Applied Biosystems CopyCaller Software v2.0. The six significant CNVs included gains (12q14.3, 15q26.3, 1p36.32, and 1p36.33) and losses (3p14.2 and 4q13.2) in NSCL/P with hypodontia patients compared with the NSCL/P only. The genes located in these regions encoded *LEMD3*, *IGF1R*, *TP73*, *SKI*, *FHIT*, and *UGT2β15*. There were a significant gain and loss of both *SKI* and *FHIT* copy number in NSCL/P with hypodontia compared with the noncleft group (*p* < 0.05). The results supported that CNVs significantly furnish to the development of NSCL/P with hypodontia.

## Introduction

Tooth agenesis (TA) is one of the most common developmental abnormalities in humans, described by the failure to develop one or more teeth (Ritwik and Patterson, 2018). TA is divided into three categories which are hypodontia, oligodontia, and anodontia (Klein et al., 2013). Hypodontia is the loss of one to five teeth, oligodontia is described as missing six or more teeth, and anodontia refers to the complete loss of tooth growth (Phan et al., 2016). Hypodontia is the most common dentofacial condition in humans (Al-Ani et al., 2017). Hypodontia can occur as an isolated condition without any other recognizable abnormality called nonsyndromic or associated with other structural deformities known as syndromic (Souza-Silva et al., 2018). The permanent dentition is more regularly affected than deciduous dentition (Abdulgani et al., 2017). A tooth development requires a complex process involving interaction between epithelial and mesenchymal signals, assisted by communication between signaling molecules and genetic pathways (Jussila and Thesleff, 2012). Various factors, such as those from wingless-related integration sites (Wnt), fibroblast growth factor (Fgf), bone morphogenic protein (BMP), and hedgehog (Hh) families, participate in the signaling mesenchymal interaction in tooth growth (Al-Ani et al., 2017). Alteration within one or more signaling pathways attributed to environmental influences (smoking, alcohol intake, chemotherapy, trauma, radiotherapy, and infection) or genetic factors could lead to abnormal tooth development (Rakhshan, 2015; Al-Ani et al., 2017).

The co-occurrence of CL/P (cleft lip with or without cleft palate) and hypodontia was reported frequently in human and animal models (Phan et al., 2016). Congenitally missing teeth or hypodontia is the most common dental anomalies found in CL/P patients (Rahman et al., 2004; Haque and Alam, 2015). Various studies revealed that *MSX1* mutations led to the cleft palate (CP) and TA in humans (Kouskoura et al., 2011; Liang et al., 2012; Leslie and Marazita, 2013). *Msx1*-deficient mice revealed severe craniofacial anomalies, comprising the secondary palatal cleft and missing teeth (Nakatomi et al., 2010). The incidence of hypodontia was higher in more severe CL/P cases and presented as loss in maxillary lateral incisor tooth (Camporesi et al., 2010). The hypodontia among CL/P may significantly impact patient’s quality of life in terms of functional decrease, emotional distress, and social prosperity.

The most common orofacial cleft syndrome is the van der Woude syndrome (VWS), which found 2% of all syndromic CL/P, caused by mutation or deletion of the *IRF6* gene in 68% of the cases (Kondo et al., 2002; de Lima et al., 2009). Letra et al. (2012) extensively investigated the role of *IRF6* in the nonsyndromic orofacial cleft (OFCs) with TA situated outside the cleft region in a cohort of 134 Brazilian patients, thereby establishing a borderline-associated *IRF6* marker (rs6588860) in the subgroup of the subjects exhibiting CP and TA. Nine genomic loci and 26 candidate genes were identified in the previous literature leading to the occurrence of those two congenital disabilities (Phan et al., 2016).

The variation in copy number has recently been identified as a significant cause of variation in the structural genome involving both duplications and sequence deletion (Redon et al., 2006). The previous study reported copy number variations (CNVs) in syndromic CL/P with hypodontia patients such as ectodermal dysplasia syndrome, Wolf Hirschhorn syndrome, and DiGeorge syndrome. The CNV in OFCs with TA found had associated with genomic loci such as in 1q21-q25, 1q32, 2q31.2-q32.2, 4p16.3, 8q24, and 16q22. However, the CNV and the related genes for nonsyndromic cleft lip with or without cleft palate (NSCL/P) with hypodontia remained with limited identification (Schinzel and Schmid, 1980; Wong et al., 1999). The causative genes and genomic loci of hypodontia among syndromic CL/P might also lead to the development in nonsyndromic CL/P. Therefore, a genome-wide association study (GWAS) was conducted to identify the contribution of CNV in the development of NSCL/P with hypodontia.

## Materials and Methods

### Study Population

A total of 81 individuals, including 61 NSCL/P cases and 20 noncleft aged 7–13, enrolled in the present comparative cross-sectional study. All the patient samples were recruited from one tertiary hospital in northern and eastern Malaysia between 2016 and 2018. The inclusion criteria for cases were NSCL/P children aged 7–13 years old, and those who had cleft palate only were excluded from this study. The subjects in the comparative group were noncleft children and those without any history of cleft. Noncleft children who have undergone orthodontic treatment were excluded from the study. The sample size for the prevalence of dental anomalies and genetic aberrations were calculated using the single proportion formula based on the prevalence of dental anomalies (Al-Kharboush et al., 2015) and CNV (Simioni et al., 2015). The precision was set at 2% giving a sample size of 42 and six subjects, respectively. However, this study recruited 61 NSCL/P patients and 100 subjects to determine the prevalence of dental anomalies. Dental panoramic tomography was taken for identification and confirmation of the number and morphology of the teeth. Exclusion criteria for the NSCL/P cases were those who had cleft palate only and patients with other syndromes such as ectodermal dysplasia or Axenfeld-Rieger syndromes. This study was approved by the Human Research Ethics Committee of Universiti Sains Malaysia (Reference number: USM/JPEPeM/140357). Both subjects and their guardians had become acquainted with the comprehensive research procedure and signed the informed consent before being enrolled in this study.

### DNA Extraction

Saliva samples were collected from each patient and stored in sterile 50-ml conical tubes. Genomic DNA was extracted from saliva samples by following GeneAll Blood SV Mini Kit manual (General Biosystem, Seoul, South Korea), including lysis, binding, washing, and elution.

### CytoScan 750K Array and Copy-Number Analysis

The Genome-Wide Human CytoScan 750K Array (Affymetrix, CA United States) was used to analyze genomic alterations according to the manufacturer’s protocol. A total of 250 ng of genomic DNA from NSCL/P with hypodontia and control samples (noncleft) were digested with the restriction enzyme *Nsp1*, then ligated to an adapter and PCR amplification using PCR on a single pair of primers that recognized the adapter sequence. The PCR products were analyzed by electrophoresis in 2% agarose using Tris-Borate-EDTA (TBE) to confirm the amplicon size between 150 and 2,000 bp in length. PCR products were combined from each sample and purified using magnetic beads (Agencourt AMPure, Beckman Coulter, Beverly, MA, United States). The purified PCR products were fragmented using DNase 1 and visualized on 4% TBE agarose gel to confirm that the fragment sizes ranged between 25 and 125 bp. The fragmented PCR products were subsequently end-labeled with biotin and hybridized to the array. The arrays were then washed, stained using GeneChips Fluidics Station 450 and Affymetrix GeneChip Command Console Software, version 1.2, followed by an Affymetrix Chromosome Analysis Suite version 3.1 (CHAS) Affymetrix United States. The data were normalized to baseline reference intensities using 270 HapMap samples and another 90 healthy noncleft in the software to calculate copy number. The Hidden Markov Model (HMM) available within the software package was used to determine the copy number states and their breakpoints. Based on HMM, the log-ratio thresholds were set at ≥0.58 and ≤−1, and used to categorize altered regions as copy number gains (amplification) and copy number losses (deletions). The alterations that only involved at least 25 conservative probes and more than 25 kbp in length were selected to avoid the false-positive CNVs.

### Candidates CNV Analysis

This study compared overlapping regions in NSCL/P and noncleft children with or without hypodontia. Hypodontia-specific CNVs were considered potential candidate variants for hypodontia among subjects if they showed a statistically significant difference between patient CNV and CNV or overlapped genes. CNVs correlate with tooth’s craniofacial production and morphogenesis or are involved in a process suspected of affecting palatogenesis and odontogenesis.

### Real-Time Quantitative PCR Validation

Two statistically significant genes were selected for validation by real-time quantitative PCR (qPCR) analysis on StepOnePlus Real-Time PCR system (Applied Biosystem, Forster City, CA, United States). The target information for the selected genes in the CNV region was entered into Assay Search Tool-Single Tube Taqman® Assay from Life Technologies website^1^ to obtain specific primer-probe pair. Two selected sequences were *Homo sapiens SKI* NCBI location Chr 2: 3,652,515-3,736,200, cytoband: 1p36.33 (Hs05780959_cn) and Fragile Histidine Triad Diadenosine Triphosphatase (*FHIT*) NCBI location Chr 3: 60555049–60579400, cytoband 3p14.2 (Hs03472126_cn). CNV performed using TaqMan Genotyping Master Mix for absolute quantitation of copy number using real-time qPCR. *RNase P* was used as endogenous control and NTC (reaction mixture without DNA template) as a negative control. qPCR was run with the following parameters: hold at 95°C for 10 min, followed by 95°C for 15 s and 60°C for 1 min for 40 cycles.

CNC for the gene is identified with Applied Biosystems CopyCaller Software v2.0. Several copies of the target sequence expected in the majority of samples were set at two. Samples were revised to attain optimum experimental conditions where samples are of high quality, copy number and reference assay have amplified, and sample replicates have parallel cycle threshold (CT) and difference of CT (∆CT) values. The number of copies of the target sequence in every test sample is regulated by relative quantitation (RQ) using the comparative CT (*Δ*ΔCT) method. This method quantifies the Ct difference (ΔCt) between target and reference sequences, then compares the ΔCt values of test samples to a calibrator sample known to have two copies of the target sequence. Accepted copy number calls should have a confidence value >95% and Z-score <1.75 (Applied Biosystems CopyCaller Software v2.0).

### Statistical Analysis

The analysis was carried out using SPSS, version 26.0. The frequency of the deletion or amplification of each CNV regions was compared between NSCL/P and noncleft with or without hypodontia using Fisher’s exact test to identify significant chromosome alterations. Chi-square tests were used to test the significant changes detected by CytoScan 750K Array. Statistical analysis for copy number data was performed using the Mann-Whitney test, and *p* < 0.05 was identified as statistically significant.

## Results

### Genome-Wide Assessment of CNVs in NSCL/P and Non-cleft With and Without Hypodontia

A total of 81 subjects were recruited in this study, including 61 NSCL/P patients and 20 noncleft subjects. In this NSCL/P, 31 (50.0%) were unilateral cleft lip and palate (UCLP), 13 (21%) were bilateral cleft lip and palate (BCLP), and 17 (27%) were cleft lip only (CL). LUCLP (34%) was the most common cleft type followed by BCLP (21.0%), RUCLP (16.0%), left cleft lip (14.0%), and right cleft lip (13.0%). The mean (SD) age for UCLP, BCLP, CL, and noncleft was 9.3 years (SD 1.8), 9.2 years (2.0), 9.1 years (2.0), and 5.5 years (3.5). Salivary samples from all subjects aged between 7 and 13 years old were subjected to genetic aberration assay using CytoScan 750K array. The subjects comprised four groups, such as NSCL/P with or without hypodontia and noncleft with or without hypodontia. All the samples exhibited chromosomal aberrations. The size of the detected CNVs varied from 25 kb to 2.5 Mb. Of the 721 genomic segments on the 81 samples, the analyses restricted to 558 genomic loci corresponding to the autosomes, including 196, showed amplification, and 362 showed deletion. The current study showed that the highest percentage of amplification occurred in chromosomes 1p, 2q, 12q, and 15q, and deletions observed for chromosomes 1q, 3p, 4q, 6p, and 7q among NSCL/P and noncleft with hypodontia. Identifying a candidate gene for NSCL/P with hypodontia in the CNVs selected regions by searching for copy number losses or copy number gains which involved the genes shared by multiple patients. The most recurrent amplified regions observed on chromosome 1p36.33, 1p36.32, 12q14.3, 14q32.33, and 15q26.3 loci and the most frequent deletion is found on chromosome 1q44, 3p14.2, 4q13.2, 6p25.3, and 7q34 among NSCL/P and noncleft with hypodontia.

Six of these unique candidate NSCL/P with hypodontia-specific CNVs (four gains and two losses) were significant, and these CNVs involved a total of eight genes. The six significant CNVs in NSCL/P with hypodontia included four gains (1p36.32, 1p36.33, 12q14.3, and 15q26.3) and two losses (3p14.2 and 4q13.2). The two significant CNVs among noncleft with hypodontia included one gain (1p36.32) and one copy number loss (4q13.2). The fisher exact analysis of the association between NSCL/P subjects in CNVs (gains and losses) are shown in Table 1. A total of six of these 334 CNVs were significantly different from NSCL/P only (*p* < 0.05). The significant CNVs included four gains (1p36.32, 1p36.33, 12q14.3, and 15q26.3) and two losses (3p14.2 and 4q13.2) and were found among NSCL/P with or without hypodontia. Besides, a total of 31 CNVs found in the noncleft with hypodontia did not overlap with the number of CNVs among noncleft-only children. Two of these were significantly different from a noncleft-only sample (*p* < 0.05) and are shown in Table 2. All the genomic loci that lay within the significant CNVs were employed to identify the phenotype like plausibly pathogenic sequence and genes of interest using Decipher database, OMIM, and Database of Genomic Variants. Table 3 shows several protein-coding genes identified from the overlapping on the significant copy number gains and losses. Two selected candidates’ genes were chosen for validation, *SKI* and *FHIT*, based on the literature (Vieira et al., 2007; Bliek et al., 2008) that the gene pathways were related to any tooth abnormalities, craniofacial deformities, and embryonic development.

**Table 1:** Association between NSCL/P with or without hypodontia with copy number variations (CNVs).

Variables	NSCL/P with hypodontia	NSCL/P without hypodontia	*X*^2^ statistic (*df*)	*p* value
	No. (%)	No. (%)		
	*n* = 41	*n* = 20		
CNVs				
Gain 1p36.32				
Gain	16 (39.0)	2 (10)	5.4 (1)	0.020
No gain	25 (61)	18 (90)		
Gain 1p36.33				
Gain	12 (29.3)	1 (5.0)	4.7 (1)	0.030
No gain	29 (70.7)	19 (95.5)		
Gain 12q14.3				
Gain	13 (31.7)	1 (5.0)	5.4 (1)	0.024
No gain	28 (68.3)	19 (95.0)		
Gain 15q26.3				
Gain	12 (29.3)	1(5.0)	4.7 (1)	0.030
No gain	29 (70.7)	19 (95)		
Loss 3p14.2				
Loss	9 (22.0)	0 (0.0)	5.4 (1)	0.024
No loss	32 (78.0)	20 (100.0)		
Loss 4q13.2				
Loss	14 (34.1)	1 (5.0)	5.8 (1)	0.023
No loss	27 (65.9)	19 (95.0)		

**Table 2:** Association between noncleft with or without hypodontia with copy number variations (CNVs).

Variables	Noncleft with hypodontia	Noncleft without hypodontia	*X*^2^ statistic (*df*)	*p* value
	No. (%)	No. (%)		
	*n* = 10	*n* = 10		
CNVs				
Gain 1p36.32				
Gain	5 (50.0)	0 (0.0)	6.7 (1)	0.033
No gain	5 (50.0)	10 (100.0)		
Gain 1p36.33				
Gain	2 (20.0)	0 (0.0)	2.2 (1)	0.474
No gain	8 (80.0)	10 (100.0)		
Gain 12q14.3				
Gain	4 (40.0)	0 (0.0)	5.0 (1)	0.087
No gain	6 (60.0)	10 (100.0)		
Gain 15q26.3				
Gain	2 (20.0)	1 (10.0)	0.3 (1)	1.00
No gain	8 (80.0)	9 (90.0)		
Loss 4q13.2				
Loss	5 (50.0)	0 (0.0)	6.7 (1)	0.033
No loss	5 (50.0)	10 (100.0)		

**Table 3:** Distribution of significant CNVs among NSCL/P and noncleft with and without hypodontia (*n* = 81).

Chr	Cytoband	CNVs	Start	End	Size (kb)	Marker count	Overlap genes	No of patients (%)
1	p36.32	Gain	329830	3615979	318	150	*TP73, PRDM16*	21 (26)
1	p36.33	Gain	849466	977958	128	70	*SKI, PRKCZ*	15 (19)
12	q14.3	Gain	66724933	66793710	69	56	*LEMD3*	18 (22)
15	q26.3	Gain	99431573	99488091	57	55	*IGF1R*	16 (20)
3	p14.2	Loss	60555048	60579400	93	38	*FHIT*	9 (11)
4	q13.2	Loss	69436558	69571630	129	25	*UGT2β15*	20 (25)

### Validation of CNVs by Real-Time qPCR

**Figure 1:**
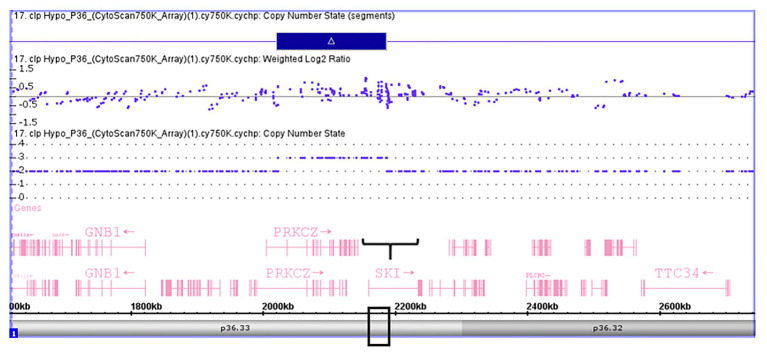
Microarray-based copy number analysis performed with the Affymetrix CytoScan 750K array and visualized using the Affymetrix Chromosome Analysis Suite version 1.2.2. Image of the 116 kb gain at 1p36.33 (Chromosome 1: 2021784-2187344, GRCH37/hg19) present in a boy 10 year-old. The region overlaps with SKI (black box).

**Figure 2:**
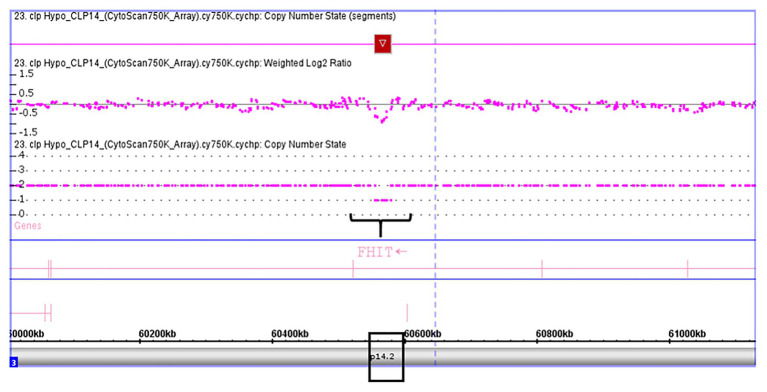
Microarray-based copy number analysis performed with the Affymetrix CytoScan 750K array and visualized using the Affymetrix Chromosome Analysis Suite version 1.2.2. Image of the 26 kb loss at 3p14.2 (Chromosome 3:60,555,048-60,579,400; GRCH37/hg19) present in a girl 9 year-old. The region overlaps with FHIT gene (black box).

Validation of *SKI* and *FHIT* by CNV assay was performed as a continuous finding of copy number gain and copy number loss found by microarray-based copy number analysis 1p36.33 and 3p14.2 regions in NSCL/P with or without hypodontia. Microarray-based copy number analysis was performed with the Affymetrix CytoScan 750K array and visualized using the Affymetrix Chromosome Analysis Suite version 1.2.2. Figure 1 shows the 116-kb gain image at 1p36.33 (Chromosome 1: 2021784–2187344; GRCH37/hg19) present in a 10-year-old boy. The region overlaps with *SKI* and *PRKCZ* genes. Figure 2 shows image of the 26-kb gain at 3p14.2 (Chromosome 3:60,555,048-60,579,400; GRCH37/hg19) present in a 9-year-old girl. The region overlaps with the *FHIT* gene.

**Figure 3:**
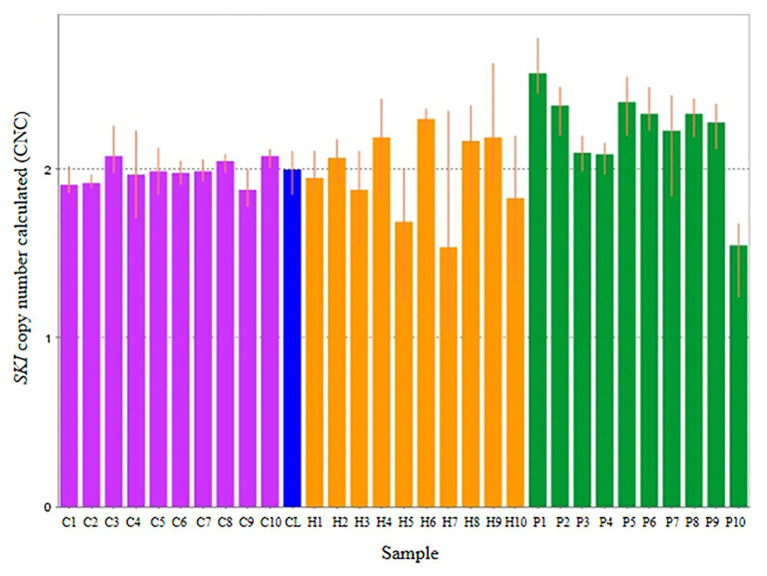
Representative of copy number calculated (CNC) of SKI in normal control (purple bars), non-cleft with hypodontia (yellow bars) NSCL/P with hypodontia (green bars) and calibrator (blue bar).

Thirty subjects were recruited, including NSCL/P and noncleft hypodontia and normal control (noncleft hypodontia). The number calculated (CNC) for *SKI* and *FHIT* genes were determined. Figure 3 showed CNC of *SKI* gene in NSCL/P with hypodontia and noncleft with hypodontia compared with the normal control. From the findings, CNC for each individual in NSCL/P with hypodontia and noncleft with hypodontia showed CNC similar to the control group, 2.49 ≥ CNC ≥ 1.50 with one patient expressing more than 2.50 CNC. The CNC in the range of 2.49 ≥ CNC ≥ 1.50 may have a copy number predicted as two. From the 30 subjects, one of the NSCL/P with hypodontia patient showed a copy number predicted (CNP) at three, and the remaining 29 samples had CNP value equal to two.

**Figure 4:**
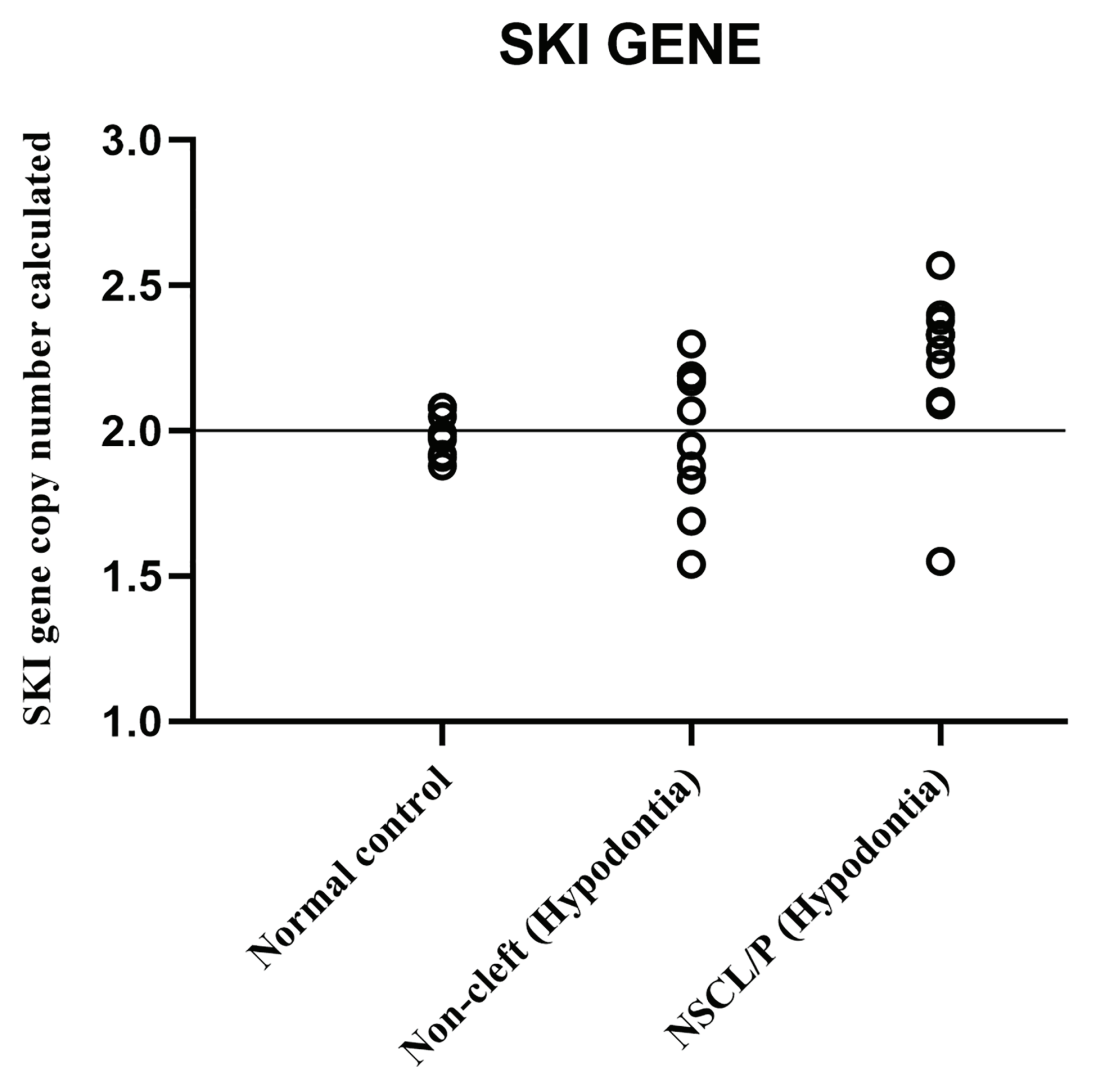
Scatter plot indicated copy number calculated value of SKI among NSCL/P with hypodontia, non-cleft with hypodontia and normal controls.

The scatter plot of the CNC data showed a distinct distribution of copy numbers between all the subjects (Figure 4). The straight line showed that the normal copy number fell at two. *SKI* copy number for NSCL/P with hypodontia was found scattered and exceeded two; meanwhile, most SKI copy numbers in normal controls were dispersed with less than copy number two. There was no significant difference in copy number of *SKI* (*p* = 0.9) in noncleft with hypodontia (1.98 ± 0.25) compared with the normal control group (1.98 ± 0.07), which means that both groups had normal copy number for *SKI*. However, there was a significant increase in *SKI* copy number in NSCL/P with hypodontia (2.2 ± 0.28, *p* = 0.02) compared with the normal control group (1.98 ± 0.07). These results revealed that *SKI* copy number gain was present among NSCL/P with hypodontia.

**Figure 5:**
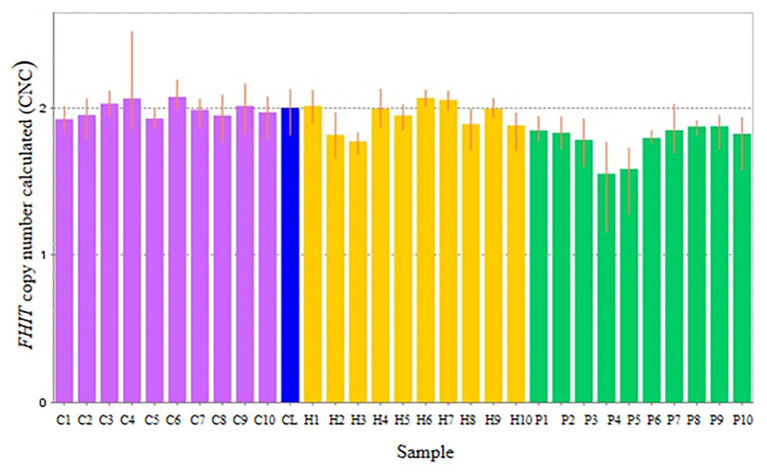
Representative of copy number calculated (CNC) of FHIT in normal control (purple bars), non-cleft with hypodontia (yellow bars) NSCL/P with hypodontia (green bars) and calibrator (blue bar).

**Figure 6:**
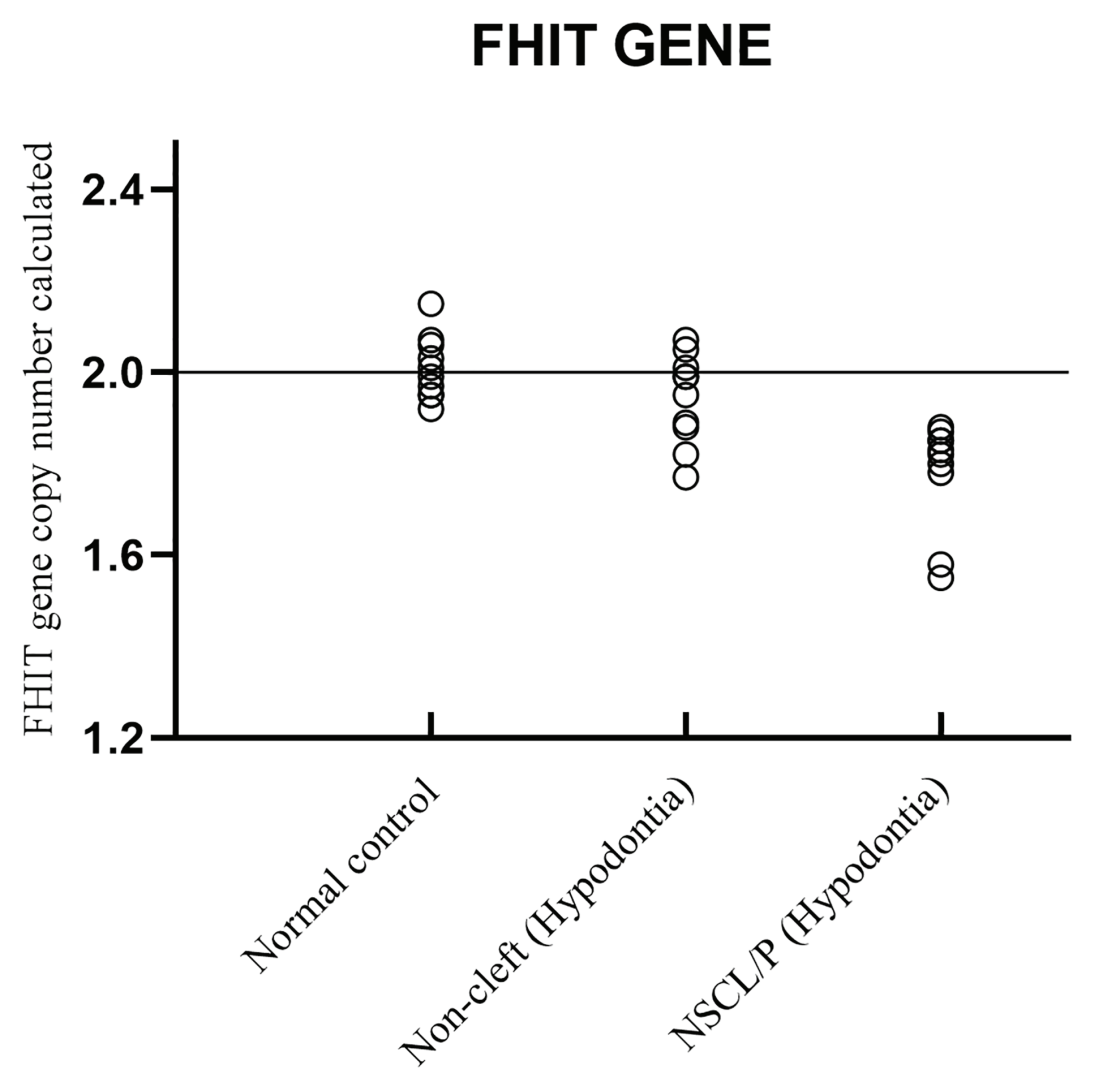
Scatter plot indicated copy number calculated value of FHIT among NSCL/P with hypodontia, non-cleft with hypodontia and normal control.

Copy number for *FHIT* was determined for NSCL/P with hypodontia as it showed copy number loss using Cytoscan 750K array. From the findings, the normal control, noncleft with hypodontia, and NSCL/P with hypodontia patients tested had a copy number of 2.49 ≥ CNC ≥ 1.50 except for two patients having CNC < 1.50 (Figure 5). Meanwhile, all the NSCL/P with hypodontia patients showed that CNC value did not exceed copy number two, including two samples with a copy number calculated as 1.55. However, scatter plot data had shown a distinct distribution of *FHIT* copy number between all the subjects (Figure 6). CNC among NSCL/P with hypodontia were lower than two compared with the normal control while noncleft with hypodontia had more copy number two. A significant decreased of *FHIT* copy number in NSCL/P with hypodontia (2.10 ± 0.231) compared with normal control (2.01 ± 0.069) was confirmed; *p* = 0.002. However, there was no significant difference in copy number of *FHIT* in noncleft with hypodontia (1.97 ± 0.09) compared with normal control.

## Discussion

### Genome-Wide Assessment of CNVs in NSCL/P and Noncleft With and Without Hypodontia

In the recent study, 61 NSCL/P and 20 noncleft children were being recruited. A total of 558 genomic loci, including 196 amplification and 362 deletions, were detected among NSCL/P and noncleft with and without hypodontia. This result showed that the copy number loss is higher compared with the copy number gain. These results are highly consistent with those previously described in a systematic analysis study by Conte et al. (2016), with a total of 249 genomic deletions and 226 duplications from a cohort of 312 orofacial clefts reported in two publicly accessible databases of chromosome imbalance and phenotype in humans, DECIPHER and ECARUCA. This study supported the finding of CNVs with small and large chromosomal deletions among oral cleft patients and individual patients with multiple congenital disabilities (Phan et al., 2016). Our study identified a significant candidate CNVs on 1p36.32, 1p36.33, 3p14.2, 4q13.2, 12q14.3, and 15q26.3 among NSCL/P patients with hypodontia whereby study done by Yu et al. (2017) reported identified 14 novel loci and genetic heterogeneity among NSCL/P only. However, we consider these CNVs important candidates because several of the genes in these regions are functionally linked to normal embryonic and tooth development. Some genomic loci with deletion were reported of TA and syndromic orofacial clefts (OFCs; Phan et al., 2016). Van Der Woude syndrome characterized as cleft lip with or without cleft palate and hypodontia revealed 8 kb deletion or insertion in the region 1q32-q41 (Schutte et al., 2000). An ectodermal dysplasia-like syndrome patient showed a 26-Mb interstitial deletion involving the region 2q31.2-q33.2, with multiple phenotypes, including missing or abnormal teeth with cleft lip and palate (Rifai et al., 2010).

Therefore, quantitative PCR was performed to confirm the data array results. The genes *SKI* and *FHIT* were randomly selected from significant CNVs (1p36.33 and 3p14.2) regions to identify their association with NSCL/P and hypodontia.

### Significant Copy Number Gain in *SKI* Among NSCL/P With Hypodontia

The present study showed a significantly increased copy number of the 1p36.33 region, encompassing the *SKI* gene. *SKI* encodes the nuclear proto-oncogene protein homolog of avian sarcoma viral (v-ski) oncogene. These findings are consistent with the previous research that showed *SKI* was the candidate gene for orofacial clefting (Berk et al., 1997); (Lu et al., 2005; Vieira et al., 2005); nevertheless, its role in tooth development remained unclear. The meta-analysis identified six new susceptible loci in European NSCL/P population including 1p36, 2p21, 3p11.1, 8q21.3, 13q31.1, and 15q22.2 (Ludwig et al., 2012). Most previous work investigated the single nucleotide polymorphism (SNP) and mutation of the *SKI* gene compared with the current study, which identified the copy number variation among the subjects. Duplication of the *SKI* gene has not yet been linked to orofacial clefting and tooth development directly. However, we found that *SKI* is involved in several signaling pathways important for the process, such as transforming factor-beta (TGF-β), BMP, and G-protein-coupled receptors (GPCR; Deheuninck and Luo, 2009; Parada and Chai, 2012).

*SKI* is an essential negative TGF-β signaling regulator, which interacts with mothers against decapentaplegic (SMADs) to suppress TGF-β signaling activity (Yang et al., 2015). *SKI* gene can block *TGF-β* signaling by interfering with the phosphorylation of SMAD2 and SMAD3 by activated *TGF-β* type 1 receptor (Buess et al., 2004). *TGF-β* signaling plays a crucial role in controlling the formation of palates in both palate epithelium and mesenchyme (Iwata et al., 2011). *TGF-β* is also critical for cell proliferation and differentiation in the dental pulp (Niwa et al., 2018). The finding may suggest that copy number gain of *SKI* may alter the building blocks of single proteins (amino acids) in the *SKI* protein. Many of the mutations modify the *SKI* protein region, which binds to SMAD proteins. It assumed that the changed *SKI* proteins could not be attached to SMAD proteins, which allow the unregulated prolongation of *TGF-β* signaling. Excessive *TGF-β* signaling affects gene activity regulation and is likely to interact with palatal and tooth growth. TGF-β3 is one of the main ligands of two serine/threonine kinase receptors, TGFβR1 and TFFβR2, investigated concerning syndromic cleft lip and palate (Loeys et al., 2005).

The *SKI* mutation was also associated with Shprintzen-Goldberg syndrome with distinctive facial features, hypotonia, and intellectual deficiency (Doyle et al., 2012; Schepers et al., 2015).

### Significant Copy Number Loss in *FHIT* Among NSCL/P With Hypodontia

A lower 3p14.2 copy number containing a *FHIT* gene was identified among NSCL/P patients with hypodontia. *FHIT* gene is a prominent member of the histidine triad gene family and is considered a tumor suppressor gene. Deleting the FHIT gene has never been reported in disturbing the development of cleft lip and palate and the tooth. Thus, this would be the first finding that revealed *FHIT* dysregulation in patients affected with NSCL/P with hypodontia. The previous result has found a deletion on the similar locus of 3p14.2 in a patient of Waardenburg syndrome type 11A with cleft lip and palate (Lakhanpal et al., 2014). Another study found that the abnormal expression of *FHIT* may be associated with a variety of malignant tumors, such as lung cancer and breast cancer (Pekarsky et al., 2002; Ismail et al., 2011). The mechanism of *FHIT* in triggering NSCL/P and hypodontia formation was unknown. However, it was likely that loss of *FHIT* is involved in the related pathway and transcriptional activities including *TGF-β*, *WNT* signaling, β-activin transcription, and *BMP* (Pekarsky et al., 2002; Weiske et al., 2007 ; Prosseda et al., 2019). This finding supports the idea that *TGF-β* signaling and canonical *Wnt/β-catenin* pathways play an essential role in the development of tooth and palatogenesis (Tamura and Nemoto, 2016; Niwa et al., 2018; Reynolds et al., 2019). There are also various reports of genetic variations at the *FHIT* locus and loss of *FHIT* protein expression in preneoplasias, proposing a tumor-suppressive role for *FHIT* in the early stages of cancer progression (Waters et al., 2014; Malak et al., 2016; Kiss et al., 2017).

Weiske et al. (2007) reported that *FHIT* related with a lymphoid enhancer-binding factor β-catenin complex by directly binding to the β-catenin, a significant player in the canonical WNT pathway that is decontrolled in numerous form of human cancer. *FHIT* expresses the transcription of target genes such as *cyclin D1*, *AXIN2*, *MMP-14*, and *survivin* when binding to the β-catenin C-terminal domain (Weiske et al., 2007). *AXIN2* likely plays a vital role in regulating beta-catenin stability in the *WNT* signaling pathway (Huraskin et al., 2016). *AXIN2* also has independently associated with tooth agenesis and NSCL/P (Letra et al., 2009). The variations in WNT genes have been recognized in an individual with tooth agenesis (Dinckan et al., 2016). Therefore, the *AXIN2* mutations could lead to an inefficient block of the *WNT* signaling pathway and altered the embryonic development of dental organs and predisposition to cancer (Otero et al., 2019). We hypothesized that reduced induction of *FHIT* might be triggering *AXIN2* mutation and directly producing *WNT* dysregulation in the progression of lip and palate fusion and tooth development and finally leading to abnormal growth.

Validation through copy number analysis has strengthened results from the previous genome-wide association studies. This current study found a significant gain and loss of copy number of these genes, *SKI* and *FHIT*, respectively, in each of the selected NSCL/P with tooth-missing patients compared with normal groups. Both genes might have a significant contribution to the development of NSCL/P and hypodontia. Different gene copy numbers among individuals and populations reveal gene expression variations (De Smith et al., 2008). CNVs also overlap over 7,000 genes, many of which are essential in biological pathways. Many studies have reported that the number of gene copies has led to a cell modification of the transcription process and make a difference in the expression level (Stranger et al., 2007; Prestel et al., 2010). Hence, evidence for CNVs between genes and potential NSCL/P with hypodontia has been confirmed in this study. Significant copy number gain of *SKI* was observed on the subject members compared with the normal control, so we can conclude that SKI might give a minor contribution to the NSCL/P with hypodontia formation and could trigger the cleft occurrence. In addition, *FHIT* loss was reported to be associated with the NSCL/P with hypodontia development. This novel finding would help to shed light on the regulation of *SKI* and *FHIT* on the orofacial cleft and hypodontia formation. In clinical setting, early complications due to hypodontia can be part of prevention strategies and genetic counseling in a multidisciplinary clinic.

## Conclusion

In conclusion, this study developed a GWAS study to explore the CNVs among NSCL/P patients and identify potential NSCL/P and hypodontia-related genes. We identified six significant CNVs with four genomic deletions and two duplication, including several genes, such as SKI, FHIT, TP73, LEMD3, IGF1R, and UGT2β15 genes. Our study enhances the reservoir of possible causative genes for NSCL/P and hypodontia genes for genetics studies. It provides a disease link to many of these genes known to contribute to several signaling pathways. Forthcoming human mutation analysis and animal model studies are required to confirm the role of the identified potential causative NSCL/P and hypodontia genes.

## Data Availability Statement

The datasets presented in this study can be found in online repositories. The names of the repository/repositories and accession number(s) can be found at: (REPOSITORY: dbVar and ACCESSION ID: nstd202).

## Ethics Statement

The studies involving human participants were reviewed and approved by Human Research Ethics Committee of Universiti Sains Malaysia (Reference number: USM/JPeM/140356). Written informed consent to participate in this study was provided by the participants’ legal guardian/next of kin.

## Author Contributions

NG performed all the experiments, designed the methodology, and wrote the original draft. NA contributed to conceptualization, formal analysis, writing-reviewing, and editing the manuscripts. AA performed the data curation and visualization. SS contributed to software analysis and validation process. TPK supervised the research and editing of the manuscript. All authors contributed to the article and approved the submitted version.

### Conflict of Interest

The authors declare that the research was conducted in the absence of any commercial or financial relationships that could be construed as a potential conflict of interest.

## Footnotes

^1^http://www.lifetechnologies.com
